# The effect of niacin on inflammatory markers and adipokines: a systematic review and meta-analysis of interventional studies

**DOI:** 10.1007/s00394-024-03425-8

**Published:** 2024-05-18

**Authors:** Esmaeil Yousefi Rad, Somayeh Saboori, Jonathan Tammam, Pariyarath Sangeetha Thondre, Shelly Coe

**Affiliations:** Oxford Brookes Centre for Nutrition and Health (OxBCNH), Department of Sport, Health Sciences and Social Work, Faculty of Health and Life Sciences, Oxford Brookes University, Gipsy Lane Campus, Headington, Oxford OX3 0BP UK

**Keywords:** Niacin, Inflammation, Adipokines, CRP, Meta-analysis

## Abstract

**Purpose:**

Niacin (nicotinic acid), known for its lipid-modifying effects, has been explored for its potential anti-inflammatory properties and potential to affect adipokines secretion from adipose tissue. The aim of this systematic review and meta-analysis was to assess the effects of niacin on inflammatory markers and adipokines.

**Methods:**

A comprehensive search was conducted across five databases: PubMed, Scopus, Cochrane Library, Embase, and ISI Web of Science. Randomized controlled trials exploring the effects of niacin on inflammatory markers (CRP, IL-6, TNF-α) and adipokines (Adiponectin, Leptin) were included. Pooled effect sizes were analysed using a random-effects model, and additional procedures including subgroup analyses, sensitivity analysis and dose-response analysis were also performed.

**Results:**

From an initial 1279 articles, fifteen randomized controlled trials (RCTs) were included. Niacin administration demonstrated a notable reduction in CRP levels (SMD: -0.88, 95% CI: -1.46 to -0.30, *p* = 0.003). Subgroup analyses confirmed CRP reductions in trials with intervention durations ≤ 24 weeks, doses ≤ 1000 mg/day, and elevated baseline CRP levels (> 3 mg/l). The meta-analysis of IL-6 and TNF-α revealed significant TNF-α reductions, while IL-6 reduction did not reach statistical significance. Niacin administration also substantially elevated Adiponectin (SMD: 3.52, 95% CI: 0.95 to 6.1, *p* = 0.007) and Leptin (SMD: 1.90, 95% CI: 0.03 to 3.77, *p* = 0.04) levels.

**Conclusion:**

Niacin treatment is associated with significant reductions in CRP and TNF-α levels, suggesting potential anti-inflammatory effects. Additionally, niacin positively influences adipokines, increasing Adiponectin and Leptin levels. These findings provide insights for future research and clinical applications targeting inflammation and metabolic dysregulation.

**Supplementary Information:**

The online version contains supplementary material available at 10.1007/s00394-024-03425-8.

## Introduction

Inflammation is increasingly associated with elevated risks of metabolic disorders, cardiovascular disease (CVD), oxidative stress and various health-threatening conditions, including cancer [1–3]. Inflammatory cytokines like interleukin-6 (IL-6) and tumour necrosis factor-alpha (TNF-α) can induce acute phase C-reactive protein (CRP) activation in the liver, and elevated levels of CRP, IL-6, and TNF-α are associated with an increased risk of CVD [4, 5].

Several human studies have demonstrated the positive effect of dietary interventions on inflammatory markers [6–8]. While Vitamin B3 (niacin or nicotinic acid) is well-known for its effectiveness in lowering triglycerides and LDL-C levels and raising HDL-C levels [9, 10], several research studies have also investigated its potential anti-inflammatory properties through randomized clinical trials.

Lee et al. conducted a one-year randomized controlled trial (RCT) where they found that administering high-dose modified-release nicotinic acid led to a considerable decrease in C-reactive protein (CRP) levels in patients with type 2 diabetes and cardiovascular disease [11]. Although another study confirmed this reduction effect [12], multiple other studies have indicated that niacin may not reduce inflammatory markers in patients consuming this vitamin [13–15].

Due to the impact in the regulation of blood lipid levels, niacin has the capacity to influence adipose tissue lipolysis and metabolism [16]. Understanding the impact of niacin on adipokine regulation can offer valuable insights into developing targeted therapeutic approaches for individuals at risk of metabolic dysregulation and related complications. Leptin and Adiponectin play pivotal roles in regulating various aspects of human metabolism, including appetite control, energy balance, insulin sensitivity, and inflammation, making them crucial factors in maintaining overall health [17]. Several studies have investigated the effects of niacin on these two important adipokines, which concluded that this treatment could beneficially increase adipokines levels [11, 18, 19]. However, there is currently no systematic review and meta-analysis to evaluate this effect.

Given the inconsistencies among studies examining the anti-inflammatory effects of niacin and its derivatives and the absence of a systematic review and meta-analysis on its impact on inflammatory markers and adipokine levels, we aimed to assess the effects of niacin on inflammatory markers (CRP, IL-6, TNF-a) as well as blood levels of Adiponectin and Leptin in a systematic review and meta-analysis on clinical intervention trials.

## Materials and methods

This systematic review and meta-analysis followed the PRISMA guidelines for systematic reviews and meta-analyses [20]. The review was registered in PROSPERO with the reference number CRD42023440132.

### Search strategy

A comprehensive search of five online databases, including PubMed, Scopus, Web of Science, Embase, and Cochrane Library, was performed, encompassing all publications from their inception until July 2023. The search strategy involved utilizing the following keywords: (niacin OR “nicotinic acid” OR “acipimox” OR niaspan) AND (“Inflammation” OR “inflammatory” OR “Tumor necrosis factor” TNF-α OR TNF OR “C-Reactive protein” OR “c reactive protein” OR “high-sensitivity CRP” OR hs-CRP OR CRP OR hsCRP OR hs-CRP OR “Cytokine” OR “Interleukin” OR “IL-6” OR “adiponectin” OR “leptin”) AND (Intervention OR “Intervention Study” OR “Intervention Studies” OR “controlled trial” OR randomized OR randomized OR random OR randomly OR placebo OR “clinical trial” OR Trial OR “randomized controlled trial” OR “randomized clinical trial” OR RCT OR blinded OR “double blind” OR “double blinded” OR trial OR “clinical trial” OR trials OR “Pragmatic Clinical Trial” OR “Cross-Over Studies” OR “Cross-Over” OR “Cross-Over Study” OR parallel OR “parallel study” OR “parallel trial”) (Table S1). There were no restrictions on language or time during the search process. All identified studies were imported into the EndNote software version 20, and duplicate citations were eliminated. The titles and abstracts of the remaining studies from the initial search underwent evaluation, and eligible studies were thoroughly reviewed in full text. Furthermore, the reference lists of relevant studies were manually checked. The literature search and screening procedures were carried out independently by two investigators.

### Inclusion and exclusion criteria

The study selection process included randomized controlled trials in adult participants 18 years or older. The trials investigated the impact of various forms of niacin administration on blood levels of CRP, IL-6, TNF- α, Adiponectin, and Leptin. The RCTs must provide mean and standard deviations (SDs) of the above markers for both the treatment and placebo groups at the beginning and end of the intervention. The selection process followed the PICO framework, encompassing the following elements: Participants (adults ≥ 18 years), Intervention (niacin), Comparison (placebo or no intervention group), and Outcomes (blood levels of CRP, IL-6, TNF- α, Adiponectin, and Leptin).

Exclusion: in vitro studies, experimental or ecological studies, observational papers, and review articles. Trials without a placebo or control group were also omitted. Additionally, studies with a two-arm intervention involving different durations or dosages were treated as separate entities during the selection process.

### Data extraction

Data extraction was performed by two independent investigators (ES & SS). If discrepancies occurred, consensus was reached through discussion. The relevant information from each study was extracted as follows: first author’s name, publication year, participants’ gender and mean age, study design, country of origin, sample sizes for both control and intervention groups, niacin dosage, type of niacin, type of control intervention, intervention duration, health status, and disease conditions of the studied population. Mean changes and standard deviations (SDs) of outcomes of trials were extracted for both the intervention and control groups. In cases where numerical estimates were presented graphically, the plot digitizer tool (http://plotdigitizer.sourceforge.net/) was utilized to accurately extract the data.

### Quality assessment

The Cochrane quality assessment Risk of bias 2 tool (ROB-2) was utilized to evaluate the potential risk of bias. This tool consists of five domains, encompassing aspects such as bias arising from the randomization process, bias due to deviations from intended interventions, and bias due to missing outcome data, measurement of the outcome, and the selection of the reported result [21]. . The risk of bias assessment was performed independently by two reviewers to ensure objectivity and rigor in the evaluation process.

### Statistical analysis

The overall effect sizes of the outcomes for both the niacin and control groups were computed using the mean changes and SDs. In instances where mean changes were not reported, we derived them based on the changes in outcome levels during the intervention. To ensure consistency, we converted standard errors (SEs), 95% confidence intervals (CIs), and interquartile ranges (IQRs) to SDs, following the method described by Hozo et al. [22].

For the analysis, a random-effects model was used, which takes into account between-study variations. Effect sizes for the variables were expressed as standardized mean differences (SMD) with their respective 95% confidence intervals (CIs) in Forest plot. To assess heterogeneity, the I^2^ statistic and Cochrane’s Q test were used. A value of I^2^ greater than 50% or a p-value less than 0.05 for the Q-test indicated significant between-study heterogeneity.

In order to investigate potential sources of heterogeneity, subgroup analyses were performed based on predefined variables. These variables included intervention duration, type of niacin used, niacin dosage, study quality, and the baseline level of CRP, due to the sufficient number of studies available for subgroup analysis. To evaluate the possibility of publication bias, an Egger’s and Begg’s regression tests were performed. Furthermore, to explore the relationship between pooled effect size and niacin dosage (mg/day) and duration of the intervention (weeks), we conducted a non-linear dose-response analysis for CRP level.

Finally, a sensitivity analysis was conducted. This analysis assessed whether the overall effect size of the meta-analysis was significantly influenced by any particular study. By systematically excluding one study at a time and re-analysing the data, we were able to gauge the impact of individual studies on the overall results. The meta-analysis was conducted using Stata, version 14 (StataCorp). A significance level of *p* < 0.05 was considered statistically significant.

## Results

### Search results and study selection

**Fig. 1:**
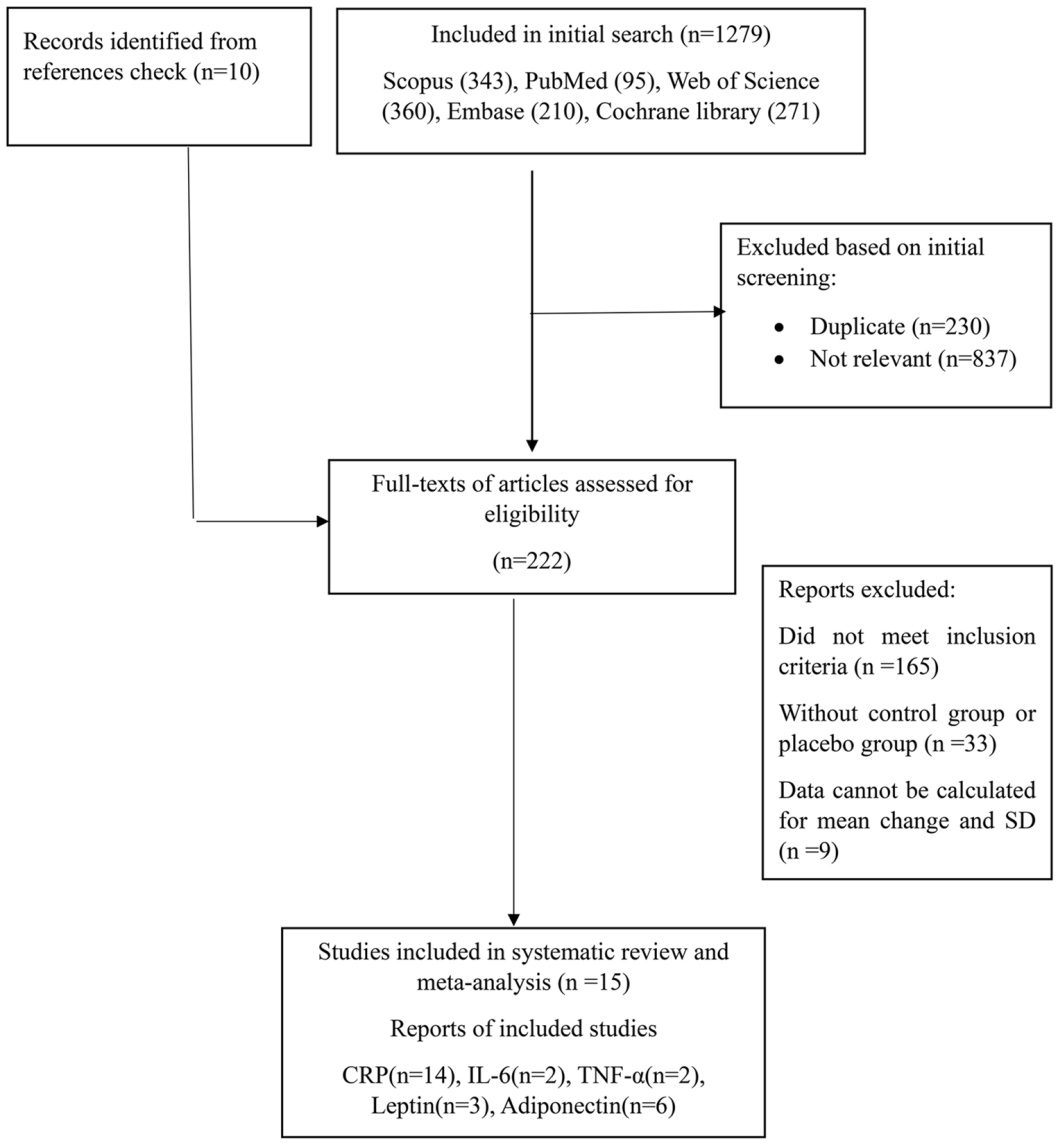
Flowchart of the study selection for inclusion in the systematic review and meta-analysis

During the initial stage of this meta-analysis, a total of 1279 publications were identified. Within this dataset, 230 articles were eliminated due to duplication, and 837 were considered unsuitable to meet the research aims, (see Fig. 1). A further 10 articles were discovered through a reference check of relevant studies. After screening of the remaining records, 222 publications were found to be eligible for full-text assessment of their eligibility.

During full-text assessment, 165 articles were excluded as they did not meet the predefined inclusion criteria. A further 33 articles were excluded as they lacked a proper control group or placebo group, while nine articles were excluded due to insufficient data for calculating the mean change and standard deviation of the mean change for our variables.

In the end, a total of 15 clinical trials were included in this systematic review and meta-analysis. Out of these studies, 14 evaluated blood levels of CRP, 2 assessed blood IL-6 and TNF- α concentration, 6 trials measured Adiponectin levels, and 3 trials assessed leptin levels. Flowchart of the study selection for inclusion in the systematic review and meta-analysis is shown in Fig. 1.

### Characteristics of the included studies

The characteristics of the randomized controlled trials (RCTs) included in this systematic review and meta-analysis are detailed in Table 1. Trials were conducted between 2003 and 2022 across regions, including the USA [15, 19, 23–26], UK [11, 27], Germany [18], Turkey [28, 29], Korea [12], China [13, 14], and Brazil [30].

**Table 1:** Summary of clinical trials on the effects of Niacin on inflammatory biomarkers and adipokines

Author, year	Country	Design	Participants	sex	Sample size	Mean ± SD age, year	Intervention		Final daily dosage (mg)	Duration (week)	Outcome
							Treatment	Control			
Wu. et at, [13]	China	RCT	Patients with vulnerable carotid atherosclerosis	Male, Female	Int: 40 Con:40	Int: 46.5 ± 6.3 Con: 45.9 ± 7.1	Acipimox + conventional treatment	conventional treatment	250	12	Hs-CRP, IL-6, TNF-a
Liu. et al., [14]	China	RCT	Patients in chronic hemodialysis	Male, Female	Int: 35 Con:37	Int:55 ± 2 Con: 56 ± 2	Niacinamide	Placebo	1500	52	CRP
Elhassan. et al., [27]	UK	Cross-over trial	Aged men	Male	Int: 12 Con:12	Median age :75 years	Nicotinamide riboside chloride (Niagen)	Placebo	1000	3	Hs-CRP, IL-6, TNF-a
Makimura. et al., [19]	USA	RCT	People with obesity	Male, Female	Int: 16 Con:15	Int:47 ± 5 Con:45 ± 7	Acipimox	Placebo	750	25.7	Adiponectin
Karacaglar. et al., [28]	Turkey	RCT	Patients with Dyslipidemia	Male, Female	Int: 25 Con:23	Int:63 ± 12 Con: 64 ± 13	Extended-release niacin+ statin	statin	500	4.2	Hs-CRP
Balasubramanyam. et al., [23]	USA	RCT	Patients with Hypertriglyceridemia	Male, Female	Int: 21 Con:24	Int:42.8 ± 1.4 Con: 44.5 ± 1.5	Sustained-release niacin (Niaspan) + low saturated-fat diet and exercise	low saturated-fat diet and exercise	2000	24	Hs-CRP, Adiponectin, Leptin
Lee. et al., [11]	Korea	RCT	Patients with intermediate coronary artery stenosis	Male, Female	Int: 14 Con:14	Int:58.1 Con: 60.8 ± 8	Niacin+ simvastatin	simvastatin	1000	38.5	Hs-CRP
Chow. et al., [24]	USA	RCT	Patients with HIV and low HDL-C	Male, Female	Int: 10 Con:9	Int:51.7 ± 4.9 Con:48.8 ± 5.1	Extended-release niacin	No intervention	1500	12	CRP
Lee. et al., [12]	UK	RCT	Patients with low HDL-C (,40 mg/dl) and either: (1) type 2 diabetes with coronary heart disease; or (2) carotid/peripheral atherosclerosis.	Male, Female	Int: 22 Con:29	Int:65 ± 9 Con:65 ± 9	Modified release NA (Niaspan)	Placebo	2000	25.7	CRP, Adiponectin
										51.4	
Linke. et al., [18]	Germany	RCT	Patients with IGT	Male, Female	Int: 30 Con:30	Int: 45.8 ± 4.1 Con: 44.7 ± 3.7	Extended-release niacin	No intervention	1000	25.7	Hs-CRP, Adiponectin, Leptin
Vaccari. et al., [26]	USA	RCT	Patients with metabolic syndrome	Male, Female	Int: 33 Con:17	Int: 32 ± 5.5 Con: 36.7 ± 4.4	Controlled-release niacin	Placebo	1000	52	Adiponectin
Thoenes. et al., [25]	USA	RCT	Patients with metabolic syndrome	Male, Female	Int: 30 Con:15	Int:34.6 ± 8.1 Con:37.5 ± 9.6	Extended-release niacin	Placebo	1000	52	Hs-CRP
Benjo. et al., 2006	Brazil	RCT	Healthy adult	Male	Int: 11 Con:11	Up to 70 years old	No-flush niacin	Placebo	1500	12.8	Hs-CRP
Taylor. et al., [15]	USA	RCT	Patients with coronary heart disease	Male, Female	Int: 87 Con:80	Int:67 ± 10 Con: 68 ± 10	Extended-release niacin (Niaspan)	Placebo	1000	51.4	CRP
Osar. et al., [29]	Turkey	RCT	Patients with Type 2 diabetes	Male, Female	Int: 15 Con:15	Int:55 ± 10 Con:59 ± 8	Nicotinamide	Placebo	50 mg/kg	4.2	CRP

Con: Control group, IGT: Impaired fasting glucose, IL-6: Interleukin 6, Int: Intervention group, TNF-a: Tumor necrosis factor alpha

With the exception of two studies that exclusively focused on male participants [27, 30], all other studies included both male and female participants. The sample sizes ranged from 19 to 167 participants, resulting in a combined cohort of 940 individuals. The average age of participants across studies ranged from 32 ± 5.5 to 75 years. Niacin dosages administered in these trials varied between 250 and 2000 mg/day. Additionally, one study employed a niacin intervention of 50 mg/kgbw/day [29]. Furthermore, the intervention durations ranged from 3 to 52 weeks.

The majority of the studies employed a parallel design of interventions, with one study utilizing a cross-over design [27]. In terms of the type of niacin used, five studies administered Extended-Release niacin (ERN) [15, 18, 24, 25, 28], one study employed Modified Release niacin [11], one study used sustained-release niacin (Niaspan) [23], two employed acipomax [13, 19], and one study utilized controlled-release niacin [26]. Additionally, two studies administered nicotinamide [14, 29], two used niacin [12, 30], and one used NR chloride (Niagen) [27]. Moreover, 3 studies incorporated the main niacin intervention alongside the use of statins [12, 28] or a Low Saturated Fat Diet and Exercise [23] or conventional treatment [13].

The RCTs encompassed a diverse array of participant groups, spanning from those with impaired glucose tolerance (IGT) [18], Type 2 diabetes [29], and metabolic syndrome [25, 26], patients with dyslipidemia [23, 28], individuals undergoing chronic hemodialysis [14], individuals with CVDs [11–13, 15], HIV-infected individuals with low HDL levels [24], to those in healthy conditions [27, 30].

According to the Cochrane ROB-2, three studies were assigned a high-quality rating, indicating a low risk of bias across all domains [14, 15, 23]. Two other studies were classified as high risk of bias trials [13, 18], and the remaining studies were designated as having some concerns for risk of bias (Table 2).

**Table 2:** Methodological quality score for included studies using Cochrane ROB-2 assessment tool

Bias domain	Criteria within domain	Wu. et at, [13]	Liu. et al., [14]	Elhassan. et al., [27]	Makimura. et al., [19]	Karacaglar. et al., [28]	Balasubramanyam. et al., [23]	Lee. et al., [11]	Chow. et al., [24]	Lee. et al., [12]	Linke.et al, [18]	Vaccari. et al., [26]	Thoenes. et al., [25]	Benjo. et al., 2006	Taylor. et al., [15]	Osar. et al., 2003
Bias arising from the randomization process	Allocation sequence randomised?	PY	Y	Y	Y	Y	Y	Y	Y	Y	Y	Y	Y	Y	Y	NI
	Allocation sequence concealed?	PN	Y	Y	NI	NI	Y	NI	NI	Y	PN	NI	Y	Y	Y	NI
	Baseline imbalances suggesting problematic randomization?	NI	NI	NI	NI	NI	NI	NI	NI	NI	NI	NI	NI	NI	NI	NI
	Roughly equal proportion of participants allocated to each group?	Y	Y	Y	Y	Y	Y	Y	Y	PN	Y	PN	PN	Y	Y	Y
	Overall risk of bias	Some concerns	Low risk of bias	Low risk of bias	Some concerns	Some concerns	Low risk of bias	Some concerns	Some concerns	Some concerns	high risk of bias	Some concerns	Some concerns	Low risk of bias	Low risk of bias	Some concerns
Bias due to deviations from intended interventions	Participants aware of assigned intervention during each trial period?	PN	N	N	NI	NI	N	NI	NI	N	PY	NI	N	N	N	NI
	Trial personnel aware of assigned intervention during each trial period?	PN	N	N	NI	NI	N	NI	NI	N	PY	NI	N	N	N	NI
	Deviations from intended interventions beyond usual practice?	NA	NA	NA	NA	NA	NA	NA	NA	NA	NA	NA	NA	NA	NA	NA
	Were deviations unbalanced between the two interventions?	NA	NA	NA	NA	NA	NA	NA	NA	NA	NA	NA	NA	NA	NA	NA
	Sufficient time for carry-over effects to disappear?	NA	NA	Y	NA	NA	NA	NA	NA	NA	NA	NA	NA	NA	NA	NA
	Overall risk of bias	Low risk of bias	Low risk of bias	Low risk of bias	Low risk of bias	Low risk of bias	Low risk of bias	Low risk of bias	Low risk of bias	Low risk of bias	high risk of bias	Low risk of bias	Low risk of bias	Low risk of bias	Low risk of bias	Low risk of bias
Bias due to missing outcome data	Outcome data available for all participants randomised?	Y	Y	Y	Y	Y	Y	Y	Y	Y	Y	Y	Y	Y	Y	Y
	Are missing outcome data similar across interventions?	NI	Y	NI	Y	NI	Y	Y	NI	Y	Y	NI	Y	NI	Y	NI
	Results robust to presence of missing outcome data?	NI	Y	NI	Y	NI	Y	Y	NI	Y	Y	NI	Y	NI	Y	NI
	Overall risk of bias	Some concerns	Low risk of bias	Some concerns	Low risk of bias	Some concerns	Low risk of bias	Low risk of bias	Some concerns	Low risk of bias	Low risk of bias	Some concerns	Low risk of bias	Some concerns	Low risk of bias	Some concerns
Bias in measurement of the outcome	Were outcome assessors aware of intervention allocation?	NI	N	N	PN	PN	N	PN	PN	N	NI	PN	N	N	N	PN
	Was outcome assessment likely to be influenced by knowledge of intervention received?	NA	NA	NA	NA	NA	NA	NA	NA	NA	NA	NA	NA	NA	NA	NA
	Overall risk of bias	Some concerns	Low risk of bias	Low risk of bias	Low risk of bias	Low risk of bias	Low risk of bias	Low risk of bias	Low risk of bias	Low risk of bias	Some concerns	Low risk of bias	Low risk of bias	Low risk of bias	Low risk of bias	Low risk of bias
Bias in selection of the reported result	Reported outcome data likely selected from multiple outcome measurements in the outcome domain?	N	PN	N	PN	PN	N	N	PN	PN	N	PN	N	PN	PN	PN
	Reported outcome data likely selected from multiple analyses of the data?	N	N	N	N	N	N	N	N	N	N	N	N	N	N	N
	Reported outcome data likely selected from the outcome of a statistical test for carry-over?	NA	NA	NI	NA	NA	NA	NA	NA	NA	NA	NA	NA	NA	NA	NA
	Overall risk of bias	Low risk of bias	Low risk of bias	Low risk of bias	Low risk of bias	Low risk of bias	Low risk of bias	Low risk of bias	Low risk of bias	Low risk of bias	Low risk of bias	Low risk of bias	Low risk of bias	Low risk of bias	Low risk of bias	Low risk of bias
Overall risk of bias judgment		high risk of bias	Low risk of bias	Some concerns	Some concerns	Some concerns	Low risk of bias	Some concerns	Some concerns	Some concerns	high risk of bias	Some concerns	Some concerns	Some concerns	Low risk of bias	Some concerns

### The effect of niacin on CRP

**Fig. 2:**
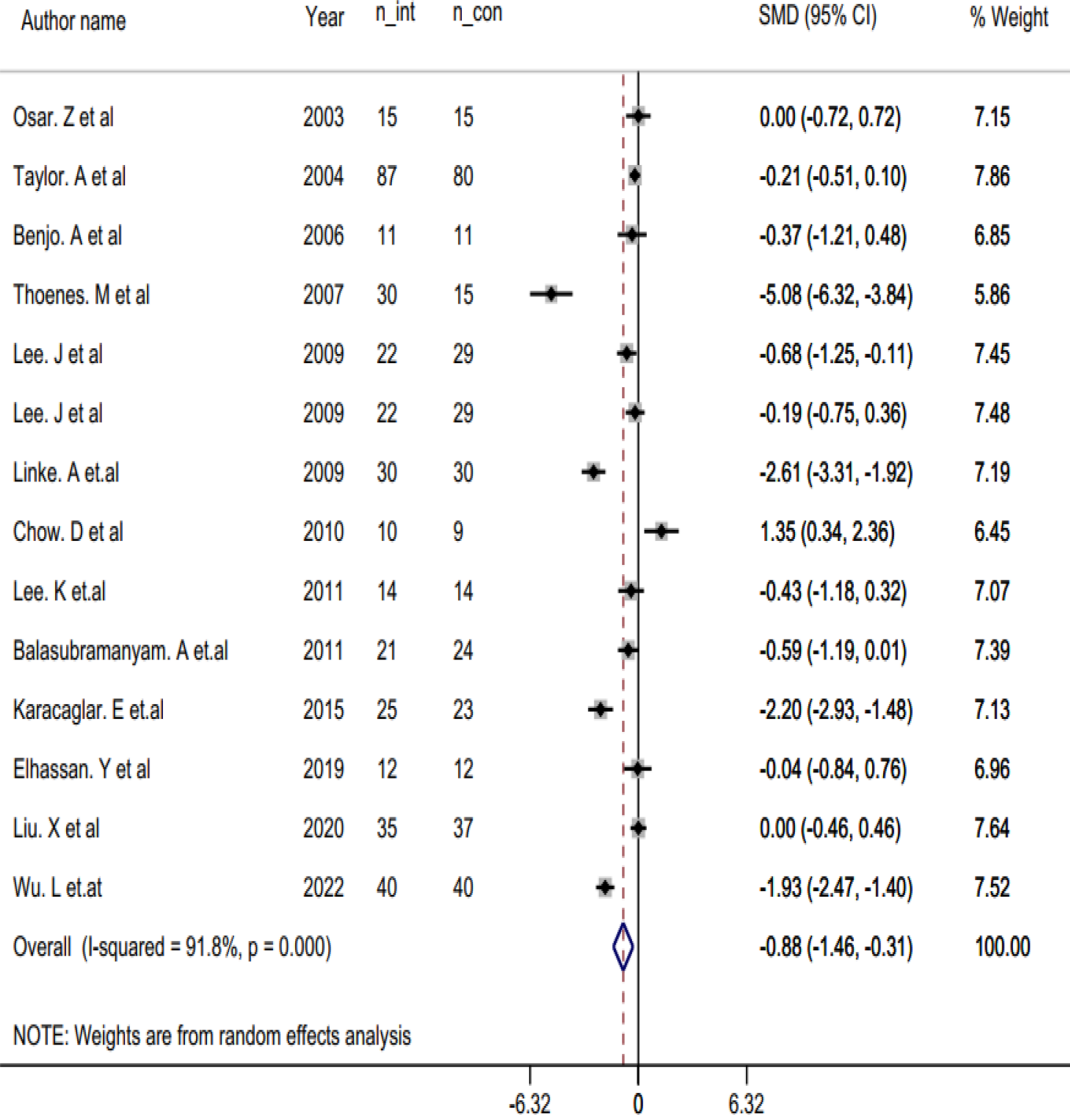
Forest plot of a random effects meta-analysis of the effect of Niacin on CRP; n_int: number of participants in intervention group, n_con: number of participants in control group

The pooled analysis of 14 effect sizes using a random-effects model revealed a significant reduction in CRP levels with the use of niacin [Standard Mean Differences (SMD): -0.88, 95% CI: -1.46 to -0.30 mg/dl, p: 0.003]. However, there was considerable heterogeneity amongst the included studies (*p* < 0.001, I^2^ = 91.8%) (Fig. 2).

To investigate potential sources of heterogeneity, we conducted subgroup analyses (Table 3). The analysis revealed a significant reduction in serum CRP concentrations with niacin intervention in several distinct cases. These include RCTs with an intervention duration of ≤ 24 weeks, trials measuring hs-CRP, and RCTs prescribing niacin at ≤ 1000 mg/day. Additionally, we observed a significant reduction in studies involving individuals with elevated CRP levels, as well as in studies administering Extended-Release niacin (ERN) or other forms of niacin.

**Table 3:** Subgroup analyses of Niacin effect on inflammatory factors and adipokines

	Number of effect sizes	SMD (95% CI)	*P*-within group	I^2^ (%)	*P*-heterogeneity	Heterogeneity between sub-groups
*Niacin effect on CRP (mg/l)*						
*Type of Niacin*						
Extended-Release Niacin (ERN)	5	-1.71(-3.37, -0.56)	0.043	96.6	< 0.001	0.048
Other forms of Niacin	9	-0.48(-0.93, -0.03)	0.034	78.5	< 0.001	
*Dosage of Niacin (mg/day)*						
≤ 1000	7	-1.72(-2.7, -0.67)	< 0.001	94.9	< 0.001	< 0.001
> 1000	7	-0.15(-0.53, 0.22)	0.43	58.8	0.02	
*Intervention duration (week)*						
≤ 24	7	-0.96(-1.95, 0.03)	0.02	90.9	< 0.001	0.105
> 24	7	-0.82(-1.56, 0.08)	0.057	93.4	< 0.001	
*hs-CRP/CRP*						
hs-CRP	8	-1.60(-2.49, -0,71)	< 0.001	91.7	< 0.001	< 0.001
CRP	6	-0.08(-0.43, 0.27)	0.65	60	0.029	
*Baseline concentrations of CRP (mg/L)*						
Normal (< 3 mg/L)	5	-0.78(-2.42, 0.86)	0.35	94.1	< 0.001	0.151
Elevated (≥ 3 mg/L)	9	-0.96(-1.55, -0.36)	0.002	91	< 0.001	
*Study quality*						
Low risk of bias	3	-0.22(-0.48, 0.05)	0.107	14.9	0.309	< 0.001
Concerns risk of bias	9	-0.80(-1.64, 0.04)	0.06	91.2	< 0.001	
High risk of bias	2	-2.24(-2.90, -1.57)	< 0.001	56.8	0.128	
*Niacin effect on IL-6*						
Overall effect	2	-1.03(-2.55, 0.48)	0.18	90.1	0.001	
*Niacin effect on TNF-a*						
Overall effect	2	-1.46(-1.89, -1.03)	< 0.001	0.0	0.61	
*Niacin effect on Adiponectin*						
Overall effect	6	4.55(2.46, 6.63)	< 0.001	96.8	< 0.001	
*Niacin effect on leptin*						
Overall effect	3	1.90(0.03, 3.77)	0.04	95.8	< 0.001	

**Fig. 3:**
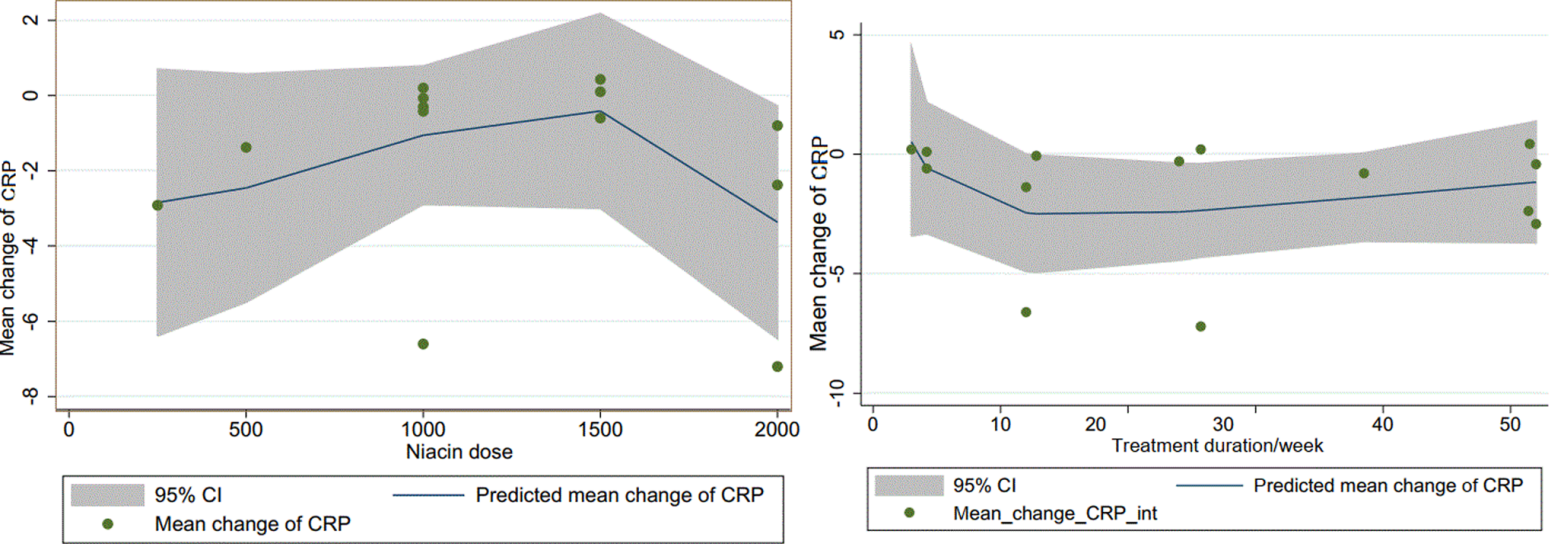
Non-linear dose–response effects of Niacin dosage (mg/day) and treatment duration on CRP. The 95% CI is demonstrated in the shaded regions

During the sensitivity analysis, we observed that removing any single study from the dataset did not substantially impact the overall estimate of the effect of niacin administration on serum CRP concentrations. The range of summary estimates remained relatively stable, ranging from − 1.61 to -0.12. Moreover, the outcomes obtained from both the Begg’s and Egger’s regression tests, indicated no notable signs of bias (*P* = 0.58 and *P* = 0.69 respectively). The dose-response analysis did not reveal any significant impact of niacin dose (P _non−linearity_ = 0.23) and treatment duration (P _non−linearity_ = 0.25) on CRP levels (Fig. 3).

### The effect of niacin on IL-6 and TNF-a

Findings from the systematic review revealed that two studies assessing serum concentrations of IL-6 and TNF-α yielded significant results. One study demonstrated a reduction effect using 1000 mg of nicotinamide riboside (NR) per day for 21 days in a placebo-controlled, randomized, double-blind, crossover trial. This reduction was observed in both serum IL-6 and TNF-α levels compared to baseline [27].

In another trial, a significant reduction was observed with the administration of 250 mg of acipomax for 12 weeks, in conjunction with conventional treatment, when compared to traditional treatment alone. This reduction was evident in both IL-6 and TNF-α levels [13].

**Fig. 4:**
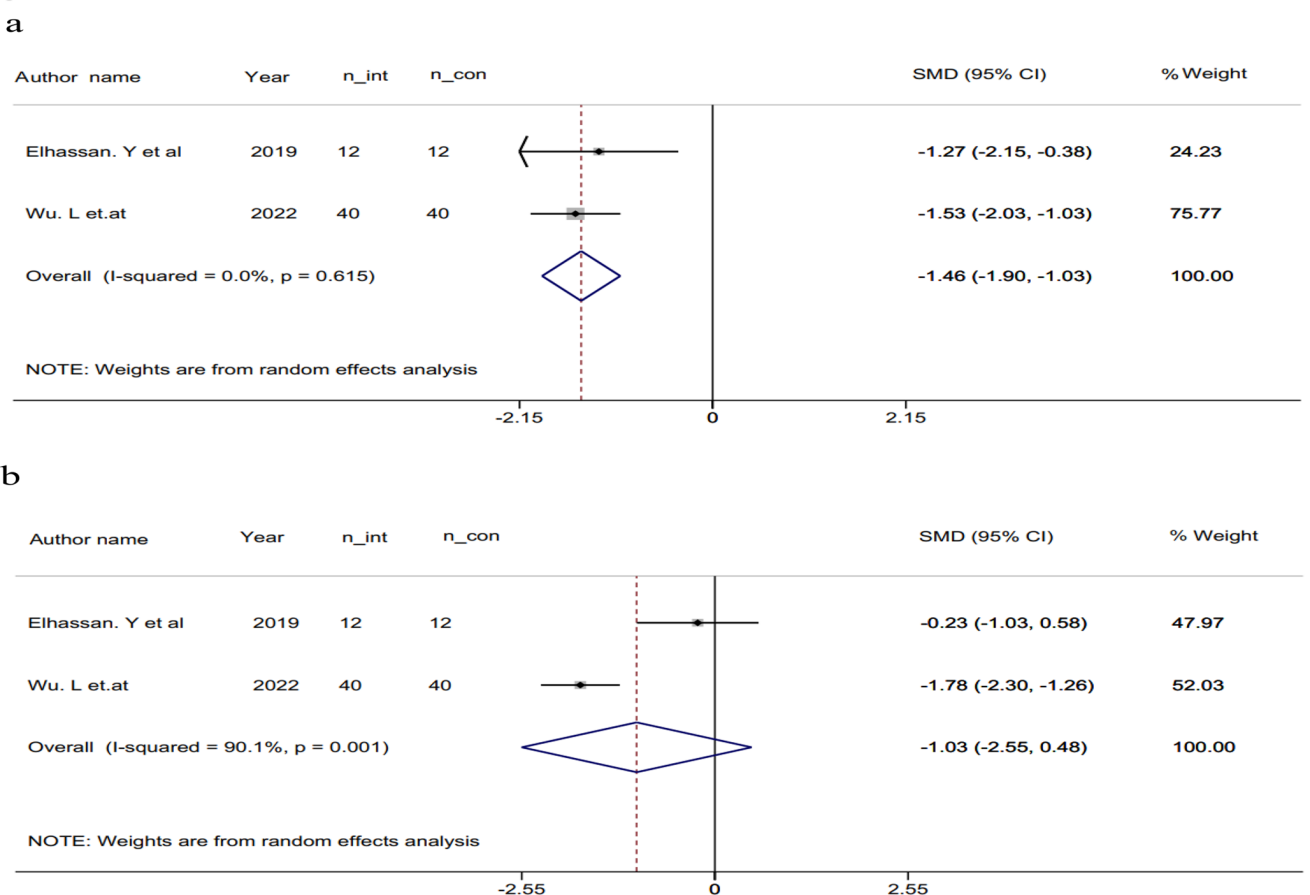
Forest plot of a random effects meta-analysis of the effect of Niacin on TNF-a (**a**) and IL-6 (**b**); n_int: number of participants in intervention group, n_con: number of participants in control group

Pooling the analysis of the two effect sizes using a random-effects model exhibited a statistically significant reduction in TNF-α levels associated with the use of niacin (SMD: -1.46, 95%CI: -1.89, -1.03, P = < 0.001, Fig. 4a). However, IL-6 concentrations did not attain statistical significance (SMD: -1.03, 95%CI: -2.55, 0.48, *P* = 0.18) (Fig. 4b).

### The effect of niacin on Adiponectin and Leptin

**Fig. 5:**
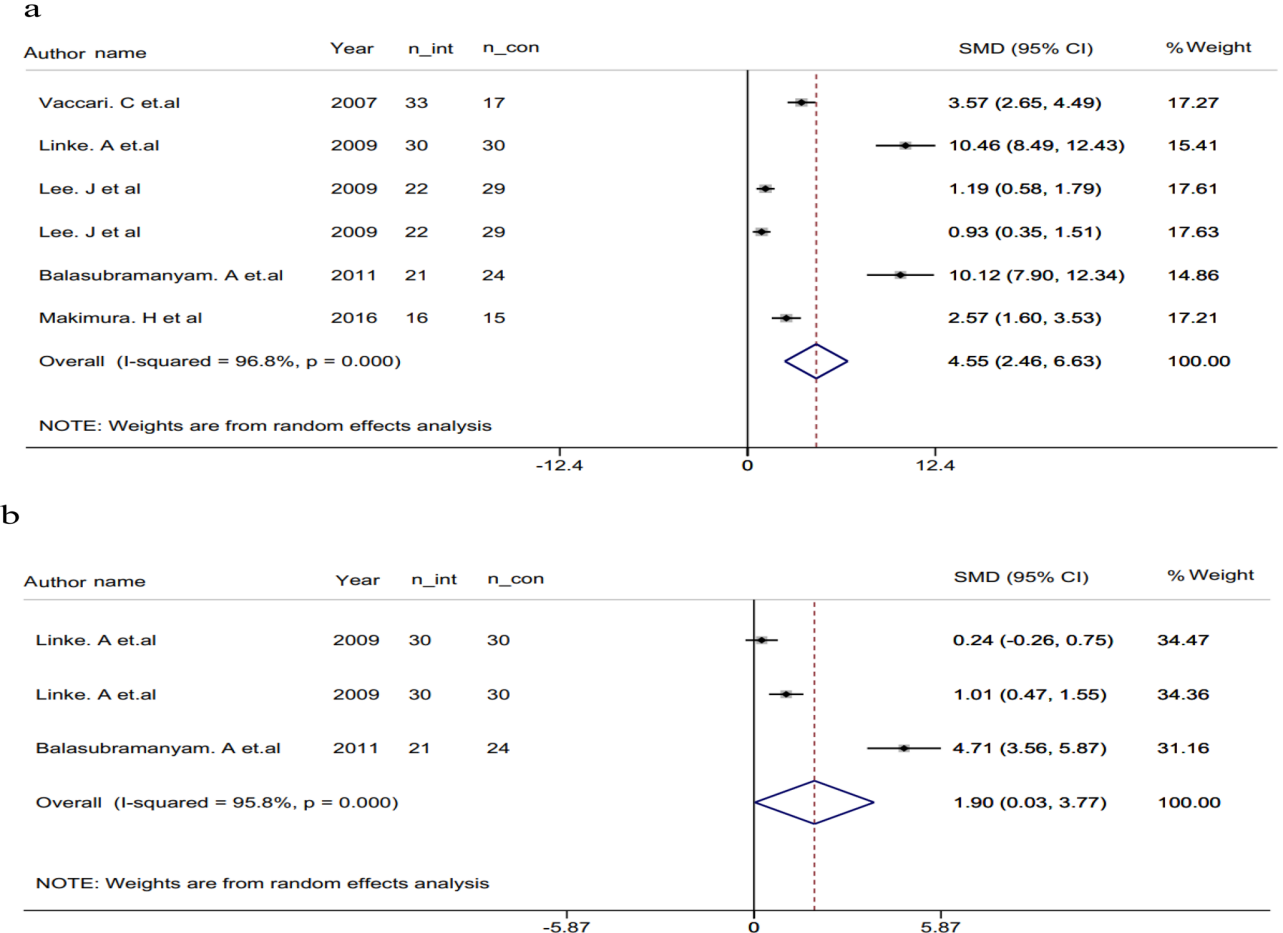
Forest plot of a random effects meta-analysis of the effect of Niacin on Adiponectin (**a**) and Leptin (**b**); n_int: number of participants in intervention group, n_con: number of participants in control group

The combined analysis of six effect sizes, utilizing a random-effects model, revealed an increase in Adiponectin levels through niacin usage SMD): 4.55, 95% CI: 2.46 to 6.63, *p* > 0.001), with significant heterogeneity across the included studies (*p* < 0.001, I^2^ = 96.8%) (Fig. 5a).

With sensitivity analysis we observed that exclusions had a minimal effect on the overall estimation of the impact of niacin administration on serum Adiponectin concentrations. The range of summary estimates remained fairly consistent, varying from 1.61 to 8.16.

Furthermore, in assessing potential publication bias through both the Begg’s and Egger’s regression tests, no significant indications of bias were observed (*P* = 0.06 and *P* = 0.26, respectively).

The analysis of three effect sizes, using a random-effects model, showed an increase in Leptin levels with the use of niacin (SMD: 1.90, 95% CI: 0.03 to 3.77, p: 0.04). There was significant heterogeneity among the included studies, as indicated by the test for heterogeneity (*p* < 0.001, I^2^ = 95.8%) (Fig. 5b). Due to the lack of included studies, conducting sub-group analysis was not possible for finding the sources of heterogeneity.

## Discussion

This systematic review and meta-analysis examined the effects of niacin on inflammatory markers and adipokines, assessing its potential as a therapeutic intervention for managing inflammation and metabolic dysfunction. The synthesis of evidence from multiple clinical trials provides valuable insights into the impact of niacin treatment on key biomarkers associated with metabolic disorders and inflammatory processes.

### Niacin and inflammatory markers

crp is a well-established marker of systemic inflammation and a predictor of cardiovascular risk [31]. The results of the current systematic review and meta-analysis showed a significant reduction in C-reactive protein (CRP) levels following niacin intervention. However, the substantial heterogeneity among the included studies highlights the need for caution in interpreting the results. The subgroup analyses revealed specific scenarios in which niacin consistently reduced CRP levels, such as baseline CRP levels more than 3 mg/dl, studies assessing hs-CRP levels, and those with niacin dosages lower than 1000 mg/day. High-sensitivity CRP (Hs-CRP) is often used to detect lower levels of inflammation and is considered a more sensitive indicator of cardiovascular risk [32]. Moreover, the significant reduction in CRP levels observed in studies involving individuals with elevated CRP levels (more than 3 mg/dl) compared to those with lower baseline CRP levels (≤ 3 mg/dl) suggests that niacin intervention might be particularly effective in individuals with existing inflammation. This could imply that niacin might have a stronger impact on reducing CRP levels in populations that are already at higher cardiovascular risk due to elevated baseline inflammation. Previous meta-analyses studies also showed that the efficacy of vitamin E, ginseng, and alpha-lipoic acid supplementation in reducing inflammatory markers was observed exclusively among individuals whose initial serum CRP levels exceeded 3 mg/dl [7, 33, 34]. These findings emphasize the importance of tailoring niacin interventions based on individual patient characteristics and needs. Furthermore, The significant reduction in CRP concentrations observed in RCTs prescribing niacin at dosages ≤ 1000 mg/day versus higher dosages (> 1000 mg/day) could indicate that there might be a threshold effect [35]. This suggests that lower dosages of niacin might be more effective in reducing CRP levels, and increasing the dosage beyond a certain point might not lead to further reductions or could even result in unexpected outcomes. Interestingly, the dose-response analysis did not identify a linear relationship between niacin dose and its impact on CRP levels, suggesting the possibility of a threshold effect for its anti-inflammatory benefits. It underscores the need for cautious interpretation of high-dosage regimens and further investigation into potential dose-dependent effects. The pooled-effect results of two studies assessing the effects of niacin on IL-6 and TNF-α revealed significant reduction in TNF-α levels while no statistically significant reduction was found in IL-6 levels.

Niacin’s anti-inflammatory effects can be attributed to a multifaceted interplay of mechanisms. By inhibiting adipose tissue lipolysis, niacin reduces the release of free fatty acids (FFAs), which are associated with inflammation and insulin resistance [36]. Through modulation of the nuclear factor-kappa B (NF-κB) pathway, niacin could inhibit the expression of pro-inflammatory genes, resulting to reduced production of pro-inflammatory cytokines IL-1β, IL-6, and TNF-α, crucial initiators of inflammation [37]. Moreover, niacin’s antioxidant properties could counteract oxidative stress, a significant contributor of inflammation, thereby suppressing inflammatory signalling pathways and reducing inflammatory marker levels [38]. Furthermore, improved endothelial function, facilitated by niacin, aids in dampening the underlying inflammatory processes, considering endothelial dysfunction’s link to inflammation and cardiovascular risk [39]. Notably, niacin’s impact on adipokines plays a role. Niacin’s ability to increase Adiponectin levels could contribute to its anti-inflammatory effects, as Adiponectin has anti-inflammatory properties [40, 41]. It’s important to note that while these mechanisms have been proposed, the exact ways in which niacin exerts its anti-inflammatory effects might involve complex interactions through various pathways. Further research is needed to fully elucidate the underlying mechanisms and to establish a comprehensive understanding of how niacin influences inflammation and inflammatory markers.

### Niacin and adipokines

This systematic review and meta-analysis showed that niacin could result in a significant increase in Adiponectin and Leptin levels. The increase in Adiponectin levels following niacin treatment suggests a potential mechanism through which niacin might improve metabolic health. However, the considerable heterogeneity observed in Adiponectin responses highlights the complexity of adipose tissue regulation and the need for further investigation into the factors influencing these responses. There are several potential mechanisms that could elucidate how niacin influences the levels of Adiponectin and Leptin. Niacin’s ability to inhibit lipolysis and reduce FFAs might lead to improved insulin sensitivity and reduced inflammation [42], indirectly affecting adipokine production while it can increase directly Adiponectin secretion by enhancing mature adipocytes through differentiation [18]. Niacin could also increase Adiponectin gene expression and secretion by its ability to activate peroxisome proliferator-activated receptor gamma (PPAR-γ), a nuclear receptor involved in adipocyte function and Adiponectin synthesis [43].

Leptin is produced primarily by adipocytes and acts as a satiety hormone, regulating energy balance [44]. Niacin’s effects on Leptin are less well-studied compared to Adiponectin. However, given niacin’s role in adipose tissue metabolism and insulin sensitivity, it could indirectly impact Leptin production. Improved insulin sensitivity can affect adipose tissue function, potentially leading to alterations in Adiponectin and Leptin secretion [41]. Furthermore, niacin’s anti-inflammatory properties might contribute to adipokine modulation. By reducing inflammation, niacin could indirectly impact Adiponectin and Leptin production [42, 45].

This study is the first systematic review and meta-analysis focusing on the impact of niacin on inflammatory markers and adipokines. However, it has some limitations, including the significant heterogeneity among the included studies. While subgroup analyses were conducted to investigate potential sources of heterogeneity for certain outcomes, the underlying causes of this heterogeneity were not identified for the other outcomes due to the limited number of studies included in the final meta-analysis. Furthermore, the included studies exhibited diversity in terms of participant populations and specific inflammatory milieu in different pathological conditions, interventions, and study designs which can affect the generalization of the findings.

In conclusion, this systematic review and meta-analysis provides valuable insights into the effects of niacin on inflammatory markers and adipokines. The observed reductions in CRP levels and increases in Adiponectin levels suggest the potential of niacin as a therapeutic agent for addressing metabolic dysregulation and inflammation. The findings underscore the need for more well-designed research to further understanding of the underlying mechanisms, optimize dosages, and refine treatment strategies. Ultimately, niacin’s potential to impact both inflammation and adipose tissue function highlights its role in the broader context of metabolic health.

## Electronic supplementary material

Below is the link to the electronic supplementary material.

Supplementary Material 1

## Data Availability

The original contributions presented in the study are included in the article/supplementary material. Further inquiries can be directed to the corresponding authors.
