# Supplementary material for: The effect of niacin on inflammatory markers and adipokines: a systematic review and meta-analysis of interventional studies
Source: Eur J Nutr. 2024 May 18;63(6):2011–24. doi: 10.1007/s00394-024-03425-8 (PMC11377601; doi:10.1007/s00394-024-03425-8)
Supplement: Supplementary file 1 — Supplementary Material 1 [file 394_2024_3425_MOESM1_ESM.docx]

**Table S1. Search strategy**

| Niacin OR "nicotinic acid" OR "acipimox" OR niaspan | Concept 1 |
| --- | --- |
| “Inflammation” OR “inflammatory” OR “Tumor necrosis factor” TNF-α OR TNF OR “C-Reactive protein” OR “c reactive protein” OR “high-sensitivity CRP” OR hs-CRP OR CRP OR hsCRP OR hs-CRP OR “Cytokine” OR “Interleukin” OR “IL-6” OR "adiponectin" OR "leptin" | Concept 2 |
| Intervention OR “Intervention Study” OR “Intervention Studies” OR “controlled trial” OR randomized OR randomized OR random OR randomly OR placebo OR “clinical trial” OR Trial OR “randomized controlled trial” OR “randomized clinical trial” OR RCT OR blinded OR “double blind” OR “double blinded” OR trial OR “clinical trial” OR trials OR “Pragmatic Clinical Trial” OR “Cross-Over Studies” OR “Cross-Over” OR “Cross-Over Study” OR parallel OR “parallel study” OR “parallel trial” | Concept 3 |
| Concept 1 AND concept 2 AND concept 3 | Search |

| 95 | ((Niacin[Title/Abstract] OR "nicotinic acid"[Title/Abstract] OR "acipimox"[Title/Abstract] OR niaspan[Title/Abstract]) AND ("Inflammation"[Title/Abstract] OR "inflammatory"[Title/Abstract] OR "Tumor necrosis factor" TNF-α[Title/Abstract] OR TNF[Title/Abstract] OR "C-Reactive protein"[Title/Abstract] OR "c reactive protein"[Title/Abstract] OR "high-sensitivity CRP"[Title/Abstract] OR hs-CRP[Title/Abstract] OR CRP[Title/Abstract] OR hsCRP[Title/Abstract] OR hs-CRP[Title/Abstract] OR "Cytokine"[Title/Abstract] OR "Interleukin"[Title/Abstract] OR "IL-6"[Title/Abstract] OR "adiponectin"[Title/Abstract] OR "leptin"[Title/Abstract])) AND (Intervention[Title/Abstract] OR "Intervention Study"[Title/Abstract] OR "Intervention Studies"[Title/Abstract] OR "controlled trial"[Title/Abstract] OR randomized[Title/Abstract] OR randomized[Title/Abstract] OR random[Title/Abstract] OR randomly[Title/Abstract] OR placebo[Title/Abstract] OR "clinical trial"[Title/Abstract] OR Trial[Title/Abstract] OR "randomized controlled trial"[Title/Abstract] OR "randomized clinical trial"[Title/Abstract] OR RCT[Title/Abstract] OR blinded[Title/Abstract] OR "double blind"[Title/Abstract] OR "double blinded"[Title/Abstract] OR trial[Title/Abstract] OR "clinical trial"[Title/Abstract] OR trials[Title/Abstract] OR "Pragmatic Clinical Trial"[Title/Abstract] OR "Cross-Over Studies"[Title/Abstract] OR "Cross-Over"[Title/Abstract] OR "Cross-Over Study"[Title/Abstract] OR parallel[Title/Abstract] OR "parallel study"[Title/Abstract] OR "parallel trial"[Title/Abstract]) | Pubmed |
| --- | --- | --- |
| 343 | ( TITLE-ABS-KEY ( niacin  OR  "nicotinic acid"  OR  "acipimox"  OR  niaspan )  AND  TITLE-ABS-KEY ( "Inflammation"  OR  "inflammatory"  OR  "Tumor necrosis factor"  tnf-α  OR  tnf  OR  "C-Reactive protein"  OR  "c reactive protein"  OR  "high-sensitivity CRP"  OR  hs-crp  OR  crp  OR  hscrp  OR  hs-crp  OR  "Cytokine"  OR  "Interleukin"  OR  "IL-6"  OR  "adiponectin"  OR  "leptin" )  AND  TITLE-ABS-KEY ( intervention  OR  "Intervention Study"  OR  "Intervention Studies"  OR  "controlled trial"  OR  randomized  OR  randomized  OR  random  OR  randomly  OR  placebo  OR  "clinical trial"  OR  trial  OR  "randomized controlled trial"  OR  "randomized clinical trial"  OR  rct  OR  blinded  OR  "double blind"  OR  "double blinded"  OR  trial  OR  "clinical trial"  OR  trials  OR  "Pragmatic Clinical Trial"  OR  "Cross-Over Studies"  OR  "Cross-Over"  OR  "Cross-Over Study"  OR  parallel  OR  "parallel study"  OR  "parallel trial" ) ) | Scopus |
| 210 | (niacin:ab,ti OR 'nicotinic acid':ab,ti OR 'acipimox':ab,ti OR niaspan:ab,ti) AND ('apolipoprotein a1':ab,ti OR 'apoa1':ab,ti OR 'apo a1':ab,ti OR 'apolipoprotein b':ab,ti OR 'apob':ab,ti OR 'apo b':ab,ti) AND (intervention:ab,ti OR 'intervention study':ab,ti OR 'intervention studies':ab,ti OR 'controlled trial':ab,ti OR randomized:ab,ti OR random:ab,ti OR randomly:ab,ti OR placebo:ab,ti OR assignment:ab,ti OR 'randomized controlled trial':ab,ti OR 'randomized clinical trial':ab,ti OR rct:ab,ti OR blinded:ab,ti OR 'double blind':ab,ti OR 'double blinded':ab,ti OR trial:ab,ti OR 'clinical trial':ab,ti OR trials:ab,ti OR 'pragmatic clinical trial':ab,ti OR 'cross-over studies':ab,ti OR 'cross-over':ab,ti OR 'cross-over study':ab,ti OR parallel:ab,ti OR 'parallel study':ab,ti OR 'parallel trial':ab,ti) | Embase |
| 271 | (Niacin OR "nicotinic acid" OR "acipimox" OR niaspan):ti,ab,kw AND (“Inflammation” OR “inflammatory” OR “Tumor necrosis factor” TNF-α OR TNF OR “C-Reactive protein” OR “c reactive protein” OR “high-sensitivity CRP” OR hs-CRP OR CRP OR hsCRP OR hs-CRP OR “Cytokine” OR “Interleukin” OR “IL-6” OR "adiponectin" OR "leptin"):ti,ab,kw AND (Intervention OR “Intervention Study” OR “Intervention Studies” OR “controlled trial” OR randomized OR randomized OR random OR randomly OR placebo OR “clinical trial” OR Trial OR “randomized controlled trial” OR “randomized clinical trial” OR RCT OR blinded OR “double blind” OR “double blinded” OR trial OR “clinical trial” OR trials OR “Pragmatic Clinical Trial” OR “Cross-Over Studies” OR “Cross-Over” OR “Cross-Over Study” OR parallel OR “parallel study” OR “parallel trial”):ti,ab,kw | Cochrane |
| 360 | Niacin OR "nicotinic acid" OR "acipimox" OR niaspan (Topic) AND “Inflammation” OR “inflammatory” OR “Tumor necrosis factor” TNF-α OR TNF OR “C-Reactive protein” OR “c reactive protein” OR “high-sensitivity CRP” OR hs-CRP OR CRP OR hsCRP OR hs-CRP OR “Cytokine” OR “Interleukin” OR “IL-6” OR "adiponectin" OR "leptin" (Topic) AND Intervention OR “Intervention Study” OR “Intervention Studies” OR “controlled trial” OR randomized OR randomized OR random OR randomly OR placebo OR “clinical trial” OR Trial OR “randomized controlled trial” OR “randomized clinical trial” OR RCT OR blinded OR “double blind” OR “double blinded” OR trial OR “clinical trial” OR trials OR “Pragmatic Clinical Trial” OR “Cross-Over Studies” OR “Cross-Over” OR “Cross-Over Study” OR parallel OR “parallel study” OR “parallel trial” (Topic) | Web of Sciences |

Total articles: 1279

After removal of duplicated articles: 1049
